# Predictive enrichment for the need of renal replacement in sepsis-associated acute kidney injury: combination of furosemide stress test and urinary biomarkers TIMP-2 and IGFBP-7

**DOI:** 10.1186/s13613-024-01349-4

**Published:** 2024-07-13

**Authors:** Lars Palmowski, Simone Lindau, Laura Contreras Henk, Britta Marko, Andrea Witowski, Hartmuth Nowak, Sandra E. Stoll, Kai Zacharowski, Bernd W. Böttiger, Jürgen Peters, Michael Adamzik, Fabian Dusse, Tim Rahmel

**Affiliations:** 1 Department of Anesthesiology, Intensive Care Medicine and Pain Medicine, University Hospital Knappschaftskrankenhaus Bochum, In der Schornau 23-25, 44892 Bochum, Germany; 2 Department of Anesthesiology, Intensive Care Medicine and Pain Medicine, University Hospital Frankfurt, Goethe-University Frankfurt, Frankfurt, Germany; 3 Center for Children and Adolescent Medicine, Sana Hospital Duisburg, Duisburg, Germany; 4 Center for Artificial Intelligence, Medical Informatics and Data Science, University Hospital Knappschaftskrankenhaus Bochum, Bochum, Germany; 5 Department of Anesthesiology and Intensive Care Medicine, Medical Faculty and University of Cologne, Cologne, Germany; 6 Department of Anesthesiology, Montefiore Medical Center and Albert Einstein College of Medicine, Bronx, NY USA; 7 Laveno-Mombello, Italy

**Keywords:** SA-AKI, FST, RRT, Precision medicine, Tissue inhibitor of metalloproteinases-2, Insulin-like growth factor-binding protein-7

## Abstract

**Background:**

In sepsis, initial resuscitation with fluids is followed by efforts to achieve a negative fluid balance. However, patients with sepsis-associated acute kidney injury (SA-AKI) often need diuretic or renal replacement therapy (RRT). The dilemma is to predict whether early RRT might be advantageous or diuretics will suffice. Both the Furosemide Stress Test (FST) and measurements of the urinary biomarkers TIMP-2*IGFBP-7, if applied solely, do not provide sufficient guidance. We tested the hypothesis that a combination of two tests, i.e., an upstream FST combined with downstream measurements of urinary TIMP-2*IGFBP-7 concentrations improves the accuracy in predicting RRT necessity.

**Methods:**

In this prospective, multicenter study 100 patients with sepsis (diagnosed < 48h), AKI stage ≥ 2, and an indication for negative fluid balance were included between 02/2020 and 12/2022. All patients received a standardized FST and urinary biomarkers TIMP-2*IGFBP-7 were serially measured immediately before and up to 12 h after the FST. The primary outcome was the RRT requirement within 7 days after inclusion.

**Results:**

32% (n = 32/99) of SA-AKI patients eventually required RRT within 7 days. With the FST, urine TIMP-2*IGFBP-7 decreased within 2 h from 3.26 ng^2^/mL^2^/1000 (IQR: 1.38–5.53) to 2.36 ng^2^/mL^2^/1000 (IQR: 1.61–4.87) in RRT and 1.68 ng^2^/mL^2^/1000 (IQR: 0.56–2.94) to 0.27 ng^2^/mL^2^/1000 (IQR: 0.12–0.89) and non-RRT patients, respectively. While TIMP-2*IGFBP-7 concentrations remained low for up to 12 h in non-RRT patients, we noted a rebound in RRT patients after 6 h. TIMP-2*IGFBP-7 before FST (accuracy 0.66; 95%-CI 0.55–0.78) and the FST itself (accuracy 0.74; 95%-CI: 0.64–0.82) yielded moderate test accuracies in predicting RRT requirement. In contrast, a two-step approach, utilizing FST as an upstream screening tool followed by TIMP-2*IGFBP-7 quantification after 2 h improved predictive accuracy (0.83; 95%-CI 0.74–0.90, p = 0.03) compared to the FST alone, resulting in a positive predictive value of 0.86 (95%-CI 0.64–0.97), and a specificity of 0.96 (95%-CI 0.88–0.99).

**Conclusions:**

The combined application of an upstream FST followed by urinary TIMP-2*IGFBP-7 measurements supports highly specific identification of SA-AKI patients requiring RRT. Upcoming interventional trials should elucidate if this high-risk SA-AKI subgroup, identified by our predictive enrichment approach, benefits from an early RRT initiation.

**Supplementary Information:**

The online version contains supplementary material available at 10.1186/s13613-024-01349-4.

## Background

Sepsis-associated acute kidney injury (SA-AKI) is one of the most frequent and detrimental complications of sepsis [1, 2]. Once SA-AKI is diagnosed, prognosis becomes severely compromised and poses additional challenges for fluid management [3, 4], particularly when a negative fluid balance is needed [5, 6]. Here, intensive care unit (ICU) physicians often face the question of whether to escalate conservative therapy and administer high-dose loop diuretics or to initiate renal replacement therapy (RRT) right away.

Considering the criteria for initiating RRT in AKI and determining its optimum timing the available data remains controversial [7]. Results from observational as well as controlled-randomized trials (RCTs) have demonstrated advantages of an early initiation of RRT in high-risk patients in terms of reduced mortality, reduced RRT duration, and enhanced renal recovery [8, 9]. Yet these reports have been challenging to replicate. Moreover, it became apparent in subsequent RTCs, that many patients initially planned to undergo RRT eventually failed to require it, due to unexpected recovery [10, 11]. Thus, an insufficiently tailored selection of high-risk patients with an early initiation of RRT, will inevitably lead to unnecessary RRT exposure [12]. Accordingly, there is an urgent need for a personalized approach with adequate specificity to more accurately identify high-risk patients eventually requiring RRT along the course of SA-AKI. Incorporating predictive enrichment into this strategy by selecting patients based on functional tests and specific urinary biomarkers could significantly aid in identifying patients most likely to benefit from early RRT.

A widely accepted functional assessment of the remaining excretory renal function is the Furosemide Stress Test (FST). Here, the patient receives an intravenous bolus of the loop diuretic furosemide and urine output over the next two hours is measured as the dependent variable. The FST serves as a prognosis for the need of RRT over the further course of sepsis and its resolution [13–15]. On the tissue injury side, on the other hand, urinary biomarkers such as the Tissue Inhibitor of Metalloproteinases-2 (TIMP-2) and Insulin-like Growth Factor-Binding Protein-7 (IGFBP-7), as measured in combination by the commercially available NEPHROCHECK® test, have shown substantial efficacy in early discerning the trajectory of AKI [16–19].

However, neither FST results nor urine TIMP-2*IGFBP-7 measurements singularly achieve sufficient diagnostic precision to reliably advocate for or against the initiation of RRT [20, 21]. Therefore, we tested the hypothesis that a combination of these two tests, i.e., an upstream functional FST combined with downstream analysis of TIMP-2*IGFBP-7 concentrations as markers of renal tubular injury improves the accuracy in predicting RRT necessity in SA-AKI.

## Materials and methods

### Study design and conceptual overview

In this multicentric, prospective, observational study (German Clinical Trials Register, DRKS00020212/ UTN U1111-1237-3685) patients were enrolled at three university hospitals in Germany from 02/2020 to 12/2022. The study was approved by the ethics committee of the Medical Faculty of the University of Bochum, Germany (ethics vote #19-6685) and subsequently by the local ethics committee of each participating center. The treatment of the patients was performed under the responsibility of the attending physicians and based on prevailing national and international guidelines as well as in-house standards and was not influenced by study participation. In this context, clinicians in charge of the patients were aware of the results of the FST, as this already represented the current best practice in each of the participating centers. The TIMP-2*IGFBP-7 values were not disclosed to the attending physicians during the conduct of the study. All research was conducted in accordance with the revised Declaration of Helsinki. Written informed consent was obtained from each patient or their legal representative.

### Study cohort

**Fig. 1:**
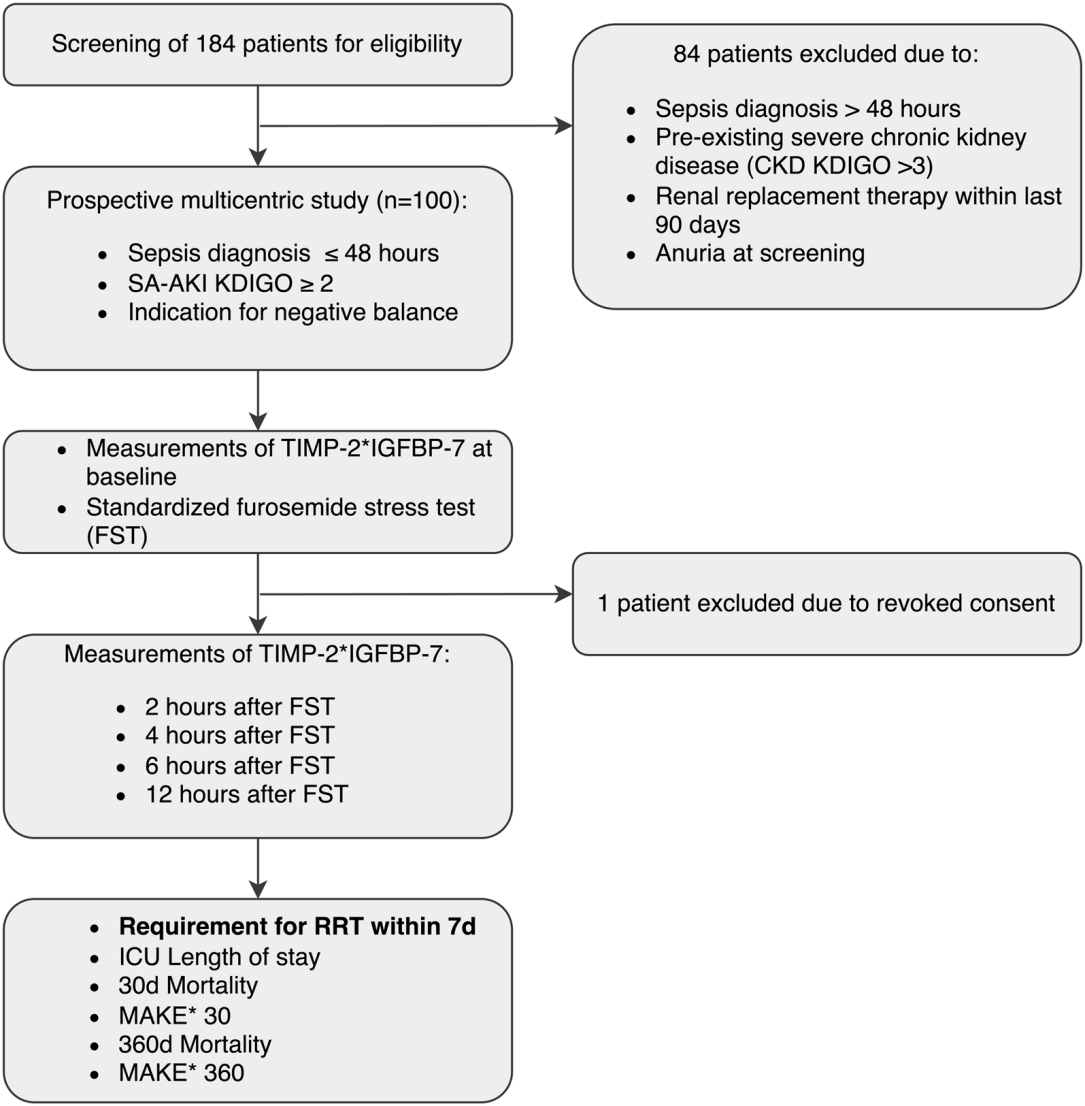
Study flow chart with inclusion criteria and sequence of conducted assessments. N = 100 patients were initially enrolled. One patient was later excluded due to revoked consent, resulting in 99 patients for final analysis. *Major adverse kidney events (MAKE) encompassing mortality, development of dialysis dependence, or the presence of persistent chronic kidney insufficiency in 30 or 360 days

We included 100 adult patients diagnosed with SA-AKI at Kidney Disease – Improving Global Outcomes (KDIGO) stage ≥ 2 within 48 h of initial sepsis diagnosis, as defined by Sepsis-3 criteria [1]. In addition, all patients had a clinical need for achieving a negative fluid balance [1, 22]. Patients with pre-existing severe chronic renal insufficiency (KDIGO > 3), prior renal transplantation (< 12 months), or a contraindication for furosemide had been excluded. Patients with anuria at enrollment were also excluded due to the inability to measure the urinary biomarkers TIMP-2 and IGFBP-7 and the obvious need for immediate RRT (Fig. 1). Regarding the indication for a negative fluid balance, the study followed the harmonized SOPs of the three participating centers. The criteria included both clinical and instrumental examinations. Clinical criteria comprised a positive cumulative fluid balance, an increase in body weight after admission by more than 5%, newly onset dyspnea without evidence of infection, and peripheral edema. Instrumental examinations included ultrasound examination of the lungs, ultrasound examination of the inferior vena cava, chest X-ray, and parameters of volume status using thermodilution technique with PiCCO. Based on the SOPs harmonized between study centers, the physicians were guided to initiate dialysis according to any of the following criteria: hyperkalemia (≥ 6 mmol/L), hyperuricemia (≥ 150 mg/dL), diuretic-resistant hypervolemia, severe metabolic acidosis (pH ≤ 7.15), oliguria (urinary output < 200 mL/24 h), or anuria.

Sample size calculation was based on a presumed incidence of 15–30% of SA-AKI patients requiring RRT [23, 24]. Based on the literature, we assumed a conservative effect size of 0.8 (Cohens D) [17, 25]. An a priori α error of 0.05 and a power of 0.80 (additionally considering a 15% safety margin) resulted in a sample size of 100 patients.

### Furosemide stress test

To avoid bias, a standardized FST was performed as follows: In patients naïve to treatment with loop diuretics, furosemide was administered intravenously at a dose of 1 mg/kg body weight. If the patient had received prior treatment with loop diuretics, the dose was increased to 1.5 mg/kg body weight. Urine volume excreted over the following two hours was measured as the dependent variable. The FST was deemed abnormal if urine volume was less than 200mL/2h [13].

### Measurements of TIMP-2 and IGFBP-7 concentration

TIMP-2 and IGFBP-7 concentrations were measured (NephroCheck® test, BioMèrieux SA, Marcy-l'Étoile, France) [26] at each test center by a trained physician and expressed as the product TIMP-2*IGFBP-7 / 1000 (AKIRisk®-Score) [27]. Measurements were made immediately before and after the standardized FST at two hours, as well as four, six, and twelve hours after furosemide application.

### Outcomes

The primary outcome was initiation of RRT within seven days of study inclusion (i.e., following the diagnosis of SA-AKI), as proposed [2]. Secondary outcomes included the length of stay (LOS) on ICU, 30-day mortality, and 360-day mortality. Additionally, the composite endpoints major adverse kidney events (MAKE) by 30 and 360 days were assessed, encompassing mortality, development of dialysis dependence, or the presence of persistent chronic kidney insufficiency (defined as a twofold increase in creatinine baseline value) [28].

### Statistics

Continuous variables are presented as means ± standard deviation (SD) in the case of normal distribution and as median and interquartile range (25th; 75th percentile) in the case of non-normal distribution of values. Group differences were examined using the t-test or Wilcoxon rank-sum test for continuous variables and the Chi-square or Fisher's exact test for categorical variables, respectively.

The cut-offs for TIMP-2*IGFBP-7 measurements were determined using the Youden Index, for both baseline and post-FST measurements. The Area Under the Curve (AUC) for predicting the need for RRT was evaluated using Receiver Operating Characteristic (ROC) analysis with just one variable, either the FST or TIMP-2*IGFBP-7 measurements. To explore the AUC when combining two variables, namely FST alongside TIMP-2*IGFBP-7 measurements, we employed a logistic regression model and analyzed the resultant Area Under the Receiver Operating Characteristic Curve (AUROC). The AUROCs were compared using the DeLong Test [29]. Reclassification analyses were performed using net reclassification improvement (NRI) to assess the added value of the combined approach compared to the isolated FST [30].

The combined approach, utilizing an upstream FST followed by downstream TIMP-2*IGFBP-7 measurement is a two-step process. Initially, the entire cohort is screened using the FST. Subsequently, abnormal results (urine output < 200mL/2h) are validated by TIMP-2*IGFBP-7 measurements after two hours, four, six, and twelve hours.

To enhance risk stratification, we conducted a comprehensive uni- and multivariable logistic regression analysis. Our analysis encompassed a range of baseline characteristics, traditional clinical chemistry renal function tests, urine output, sequential TIMP-2*IGFBP-7 measurements, and FST outcomes.

An alpha error p of less than 0.05 was considered statistically significant. The confidence interval (CI) was calculated with 95% coverage. Statistical analyses were performed using the software R (version 3.5.3; The R Foundation for Statistical Computing; http://www.R-project.org).

## Results

### Study cohort and demographics

A total of 100 patients were initially enrolled in the study. One patient subsequently revoked his consent to participate in the study, resulting in 99 patients included in the final analysis (Fig. 1). Our final cohort comprised of 57 males (58%), with a median age of 72 years (IQR: 60.0 to 79.0), and a median SOFA score of 10 (IQR: 8.0 to 12.0). Of the whole cohort, 32 patients (32%) received RRT within seven days after enrollment (p = 0.860 between centers). The patients requiring RRT demonstrated higher SOFA scores, greater lactate concentrations, and a smaller urine output following FST compared to patients not requiring RRT at baseline (each p < 0.01, Table 1).

**Table 1:** Cohort description, classification according to the implementation of RRT within the first 7 days

	Overall (n = 99)	RRT (n = 32)	No-RRT (n = 67)	p-value
**Base characteristics**				
Age, years (IQR)	72.00 (60.00–79.00)	69.50 (62.50–77.50)	75.00 (59.50–79.50)	0.637
Male sex, n (%)	57 (58%)	21 (66%)	36 (54%)	0.286
Height, cm (IQR)	177.00 (170.00–180.80)	178.00 (172.00–183.50)	175.00 (170.00–180.00)	0.113
Weight, kg (IQR)	84.90 (73.10–94.45)	89.00 (72.08–98.95)	83.60 (73.35–94.30)	0.540
Body mass index, kg/m^2^ (IQR)	26.50 (23.32–30.75)	26.00 (22.80–30.15)	26.60 (24.05–31.00)	0.786
SOFA Score, day 1 (IQR)	10.00 (8.00–12.00)	12.00 (9.50–13.50)	10.00 (8.00–11.00)	**0.001**
Ventilatory support*, day 1, n (%)	51 (52%)	18 (56%)	33 (49%)	0.528
Horowitz index, day 1 (IQR)	263.00 (197.50–368.50)	239.00 (159.20–315.50)	296.00 (201.00–369.00)	0.258
Length of ICU stay, days (IQR)	11.00 (7.00–19.50)	10.50 (6.75–15.50)	12.00 (8.00–20.50)	0.253
Urine output in FST**, mL (IQR)	264.84 (64.74–495.63)	42.76 (16.77–272.95)	355 (211.10–550.20)	** < 0.001**
Comorbid conditions, n (%)				
Hypertension	58 (59%)	23 (72%)	35 (52%)	0.082
Cardiovascular disease	54 (55%)	17 (53%)	37 (55%)	1
Chronic heart failure	8 (8%)	1 (3%)	7 (10%)	0.431
COPD***	22 (22%)	6 (19%)	16 (24%)	0.617
Diabetes mellitus	36 (36%)	12 (38%)	24 (36%)	1
Chronic kidney disease	17 (17%)	9 (28%)	8 (12%)	0.108
Malignant neoplasms	38 (38%)	12 (38%)	26 (39%)	1
Infection focus, n (%)				
Pulmonal	36 (36%)	7 (22%)	29 (43%)	0.085
Urinary tract	5 (5%)	3 (9%)	2 (3%)	
Abdomen	32 (32%)	12 (38%)	20 (30%)	
Central nervous system	7 (7%)	1 (3%)	6 (9%)	
Other/unknown	19 (19%)	9 (28%)	10 (15%)	
Laboratory values, day 1				
Potassium, mmol/L (IQR)	4.30 (4.00–5.00)	4.40 (4.10–5.10)	4.30 (4.00–4.85)	0.339
Creatinine, mg/dL (IQR)	2.21 (1.53–3.31)	2.08 (1.38–3.26)	2.27 (1.73–3.31)	0.525
Urea, mg/dL (IQR)	75.00 (48.00–117.50)	51.50 (41.50–105.00)	84.00 (53.00–121.50)	0.075
Lactic acid, mmol/L (IQR)	2.07 (1.44–3.05)	3.20 (1.99–4.05)	1.80 (1.35–2.40)	** < 0.001**
Platelets, /nL (IQR)	156.00 (111.2–225.8)	148.00 (87.50–213.00)	172.00 (116.5–234.00)	0.338
Bilirubin, mg/dL (IQR)	0.85 (0.50–2.40)	0.90 (0.55–2.40)	0.80 (0.50–2.45)	0.789
White blood cells/µL (IQR)	14.59 (8.17–22,69)	17.27 (12.06–22.70)	13.86 (6.31–22.62)	0.212
C-reactive protein, mg/L (IQR)	91.45 (46.77–211.69)	71.18 (29.96–164.50)	100.26 (52.31–212.69)	0.108
Procalcitonin, ng/mL (IQR)	6.85 (14.32)	8.61 (2.48–16.81)	6.50 (2.42–14.35)	0.357
GOT, U/L (IQR)	98.00 (43.25–139.00)	104.00 (59.00–117.00)	83.00 (33.00–141.00)	0.755
GPT, U/L (IQR)	54.00 (23.00–77.00)	55.00 (29.00–76.00)	53.50 (22.25–77.00)	0.851

Data are presented as n (%) and median (IQR)

^*^Ventilatory support included: CPAP / High flow / Invasive

^**^Urine output within 2 h after Furosemide application during furosemide stress test (FST) as described in methods

^***^Chronic obstructive pulmonary disease (COPD)

Initial creatinine (RRT 2.08 mg/dL, IQR: 1.38 to 3.26 vs. non-RRT 2.27 mg/dL, IQR: 1.73 to 3.31, p = 0.53) and urea concentrations (RRT 51.5 mg/dL, IQR: 41.5 to 105.0 vs. non-RRT 84.0 mg/dL, IQR: 53.0 to 121.5, p = 0.08) showed no statistically significant differences at enrollment. Further baseline characteristics are provided in Table 1.

Considering the secondary endpoints, the following outcomes were observed: The median ICU LOS for the cohort was eleven days (IQR: 7.0 to 19.5) and did not differ between RRT (10.5 days, IQR: 6.75 to 15.5) and non-RRT patients (12 days, IQR: 8.0 to 20.5, p = 0.25). 84 out of 99 patients (85%) experienced MAKE30, with comparable rates between the RRT (84%, 27 of 32 patients) and the non-RRT subgroup (84%, 57 of 67 patients, p = 1.0). In contrast, 68 of 99 patients (69%) encountered MAKE360, showcasing a discrepancy between RRT (91%, 29 of 32 patients) and non-RRT patients (58%, 39 of 67 patients, p < 0.01). The 30-day mortality rate was 36% (36 of 99 patients), with no statistically significant difference between the RRT (47%, 15 of 32 patients) versus the non-RRT subgroup (31%, 21 of 67 patients, p = 0.18). However, the 360-day mortality rate reached 51% (50 of 99 patients), with differences between RRT (69%, 22 of 32 patients) and non-RRT patients (42%, 28 of 69 patients, p = 0.05). Further details on these secondary endpoints are provided in Table 2.

**Table 2:** Secondary endpoints according to RRT-dependency

Endpoint	RRT (n = 32)	No-RRT (n = 67)	p-value
MAKE 30	27 (84%)	57 (85%)	1
30 day: Mortality	15 (47%)	21 (31%)	0.18
CKD	12 (38%)	36 (54%)	0.14
RRT	1 (3%)	0 (0%)	0.32
MAKE 360	29 (91%)	39 (58%)	< 0.01
360 day: Mortality	22 (69%)	28 (42%)	0.05
CKD	7 (22%)	11 (16%)	0.05
RRT	7 (22%)	3 (4%)	0.01

*Data are presented as n (%), Major adverse kidney events (MAKE), Chronic kidney disease (CKD), Renal replacement therapy (RRT)*

### Trajectories of urinary TIMP-2*IGFBP-7 measurements

**Fig. 2:**
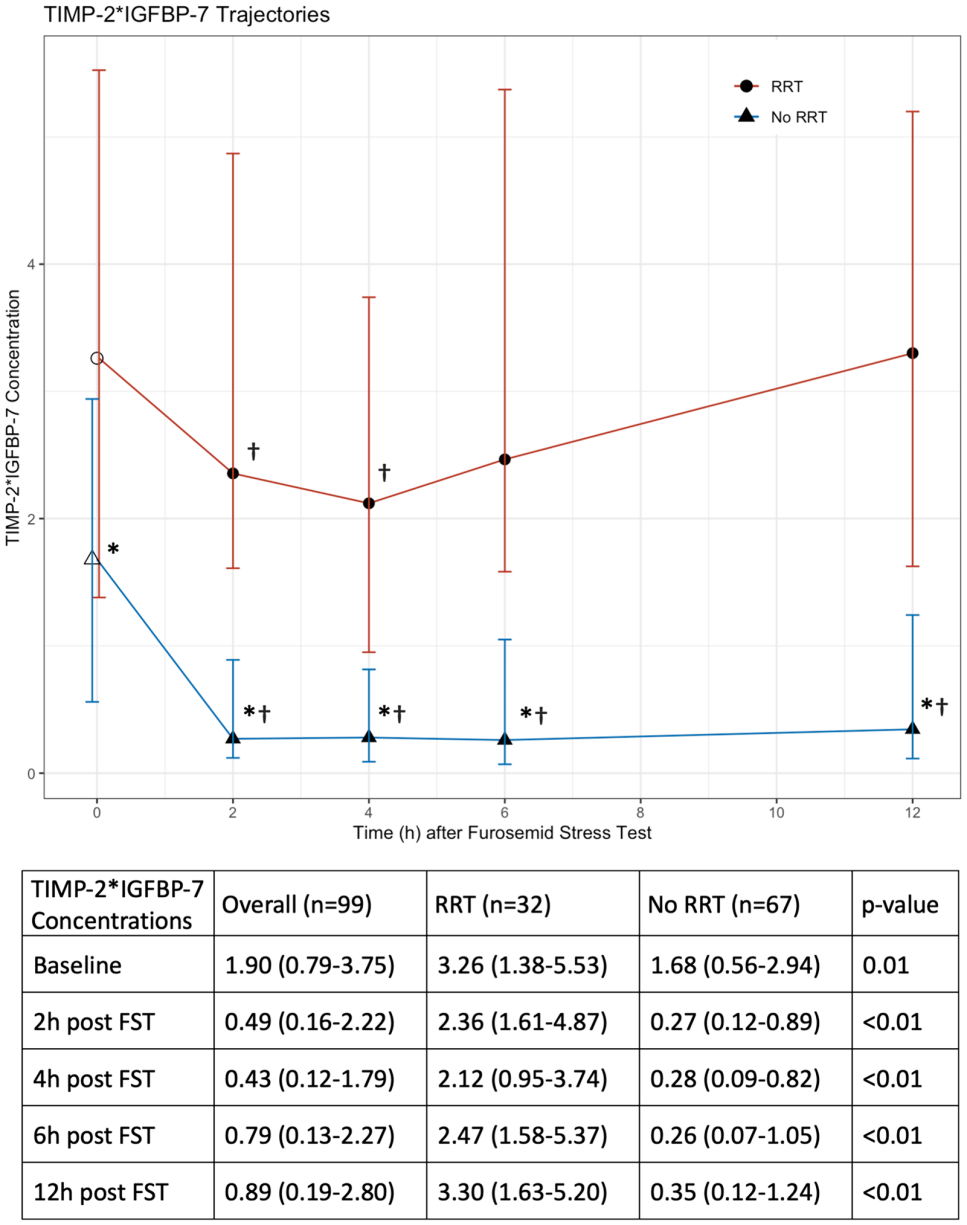
The X-axis represents the time after the FST. Measurement samples at baseline before the furosemide stress test (FST) at time point 0 h were taken immediately before the administration of furosemide. The Y-axis shows the median results of the TIMP-2*IGFBP-7 measurements (in ng^2^/mL^2^/1000) with IQR as error bar. The RRT and the non-RRT subgroup showed significant differences at each time point between both groups. While TIMP-2*IGFBP-7 concentrations in non-RRT patients decreased after FST and remained significantly lower, RRT patients reached its minimum of TIMP-2*IGFBP-7 concentrations 4h after FST with a following increase, exceeding baseline measurements after 12h. *represents statistical significance to baseline measurement before FST. † represents statistical significance between RRT and non-RRT subgroup

At baseline prior to the FST, the median urinary TIMP-2*IGFBP-7 concentrations were 1.90 ng^2^/mL^2^/1000 (IQR: 0.79 to 3.75). Concentrations were greater in patients eventually requiring RRT (3.26 ng^2^/mL^2^/1000, IQR: 1.38 to 5.53) compared to those who did not (1.68 ng^2^/mL^2^/1000, IQR: 0.56 to 2.94, p = 0.01). Two hours post-FST, TIMP-2*IGFBP-7 concentrations had decreased to a median of 0.49 ng^2^/mL^2^/1000 (IQR: 0.16 to 2.22), still with a significant distinction between RRT patients (2.36 ng^2^/mL^2^/1000, IQR: 1.61 to 4.87) and non-RRT patients (0.27 ng^2^/mL^2^/1000, IQR: 0.09 to 0.82, p < 0.01), as demonstrated in Fig. 2.

The concentrations of TIMP-2*IGFBP-7 remained lower for up to twelve hours post-FST in non-RRT patients (each time point p < 0.05). In contrast, RRT patients had their lowest TIMP-2*IGFBP-7 concentration at four hours post-FST with 2.12 ng^2^/mL^2^/1000 (IQR: 0.95 to 3.74, p < 0.01 compared to baseline), with a rebound at twelve hours to 3.30 ng^2^/mL^2^/1000 (IQR: 1.63–5.20, p = 0.71 compared to baseline). In addition, RRT patients showed higher TIMP-2*IGFBP-7 concentrations at baseline and all further time points compared to non-RRT patients (each p < 0.05, Fig. 2).

### Performance of FST for the prediction of RRT within seven days

**Fig. 3:**
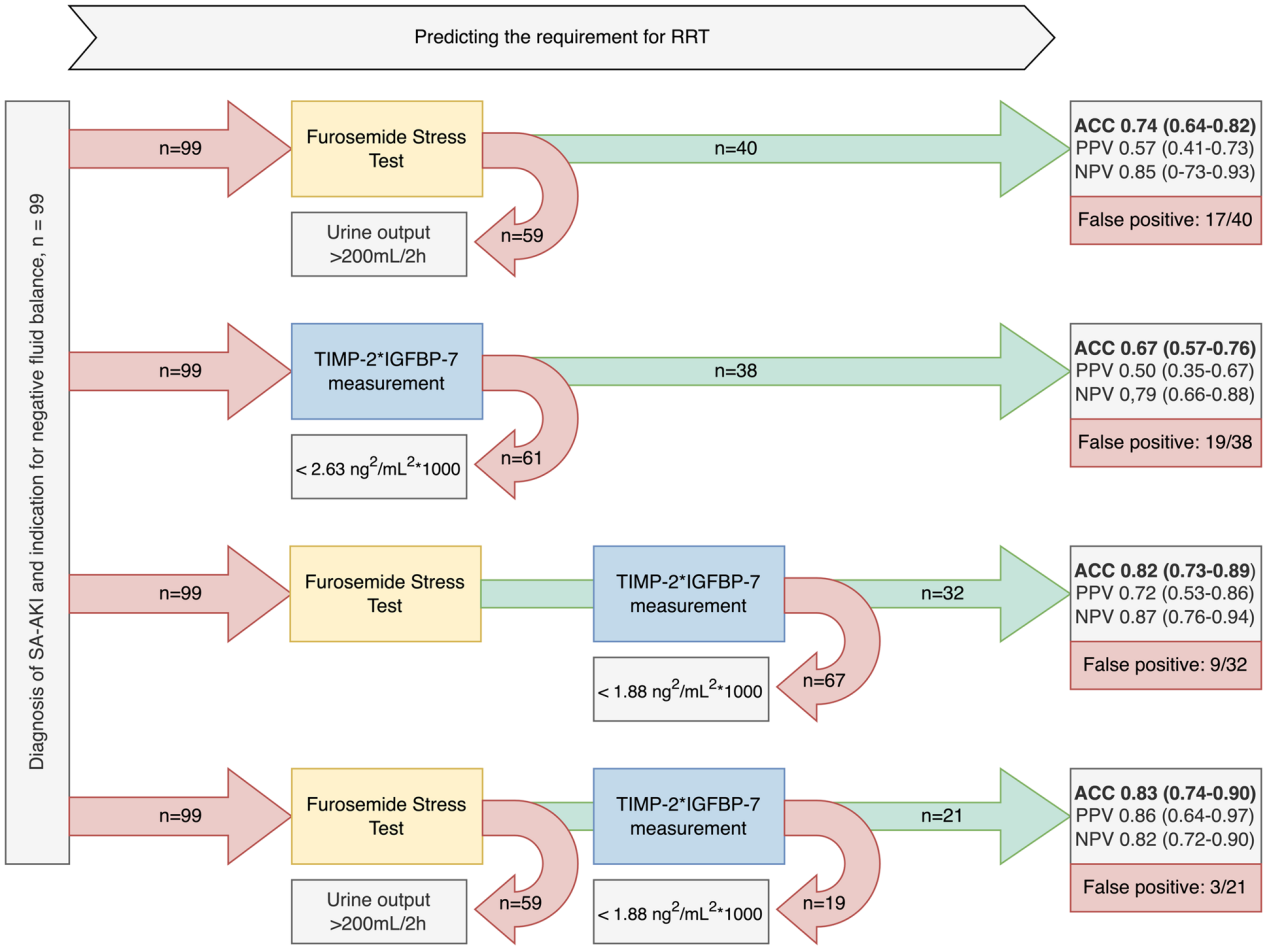
Various strategies for optimizing the prediction of RRT dependency within seven days after SA-AKI diagnosis. An enhanced accuracy (ACC) and positive predictive value (PPV) for the FST were attained by first employing the FST (with an urine output cutoff of 200mL/2h) as an upstream assessment and subsequently validating renal pathology by urine TIMP-2*IGFBP-7 measurements. However, there was no significant difference observed in the negative predictive value (NPV). The cut-offs for TIMP-2*IGFBP-7 measurements before and after the FST, specifically 2.63 ng^2^/mL^2^/1000 and 1.88 ng^2^/mL^2^/1000, were calculated based on the Youden Index, as described in the methods section

**Fig. 4:**
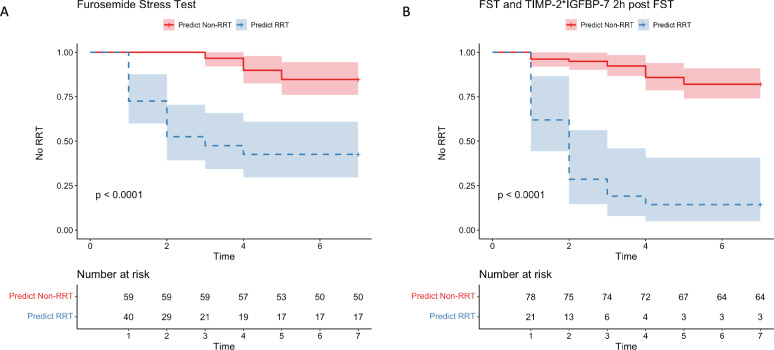
Kaplan–Meier Curve for prediction of RRT need within seven days after diagnosis of SA-AKI using solely FST (**A**) or FST and TIMP-2*IGFBP-7 measurements two hours after the FST (**B**). If the FST is used as an upstream screening tool and pathological results are confirmed by TIMP-2*IGFBP-7 measurements, 21 of 99 patients are classified as high risk for eventually developing RRT dependency. Among these 21 patients, 18 actually received dialysis within seven days

Utilizing the FST for predicting the need of dialysis within the next seven days resulted in an AUROC of 0.82 (95%-CI 0.72 to 0.91, supplementary file 1), with an accuracy of 0.74 (95%-CI: 0.64 to 0.82), a sensitivity of 0.72, and a specificity of 0.75 (Table 3, Figs. 3, 4).

**Table 3:** Test Accuracy of Furosemide Stress Test (FST) and TIMP-2*IGFBP-7 measurements

Test	AUROC	Cut-off	Accuracy	Sensitivity	Specificity	PPV	NPV
FST	0.82 (0.72–0.91)	> 200 mL/2 h	0.74 (0.64–0.82)	0.72 (0.53–0.86)	0.75 (0.63–0.84)	0.57 (0.41–0.73)	0.85 (0.73–0.93)
TIMP-2*IGFBP-7 **before** FST	0.66 (0.55–0.78)	> 2.63 ng^2^/mL^2^/1000	0.67 (0.57–0.76)	0.61 (0.42–0.78)	0.70 (0.57–0.81)	0.50 (0.33–0.67)	0.79 (0.66–0.88)
TIMP-2*IGFBP-7 **2 h after** FST	0.80 (0.70–0.90)	> 1.88 ng^2^/mL^2^/1000	0.82 (0.73–0.89)	0.71 (0.53–0.86)	0.87 (0.76–0.94)	0.72 (0.53–0.86)	0.87 (0.76–0.94)
FST **and** TIMP-2*IGFBP-7 **after 2 h**	0.84 (0.75–0.93)	> 200 mL/2 h *and* > 1.88 ng^2^/mL^2^/1000	0.83 (0.74–0.90)	0.56 (0.38–0.74)	0.96 (0.88–0.99)	0.86 (0.64–0.97)	0.82 (0.72–0.90)

TIMP-2*IGFBP-7 measurements immediately before and 2 h after FST, as well as combined evaluation of TIMP-2*IGFBP-7 and FST. Area under the receiver operating characteristic (AUROC), accuracy, sensitivity/specificity, positive predictive value (PPV) and negative predictive value (NPV), along with corresponding 95% confidence intervals

### Performance of TIMP-2*IGFBP-7 measurements for the prediction of RRT within seven days

While TIMP-2*IGFBP-7 baseline measurements (0h) immediately before the FST showed an AUROC of 0.66 (95%-CI 0.55 to 0.78, supplementary file 1) for the prediction of RRT requirement, its measurement two hours after the FST indicated an AUROC of 0.80 (95%-CI: 0.70 to 0.90, supplementary file 1), significantly outperforming the baseline measurements (p < 0.01). Utilizing the Youden Index (cutoff: 1.88 ng^2^/mL^2^/1000) for the TIMP-2*IGFBP-7 measurements at two hours post-FST demonstrated an accuracy of 0.82 (95%-CI: 0.74 to 0.90) with a sensitivity of 0.71 and a specificity of 0.87 (Table 3, Fig. 3).

### Combined assessment of FST and TIMP-2*IGFBP-7 measurements for prediction of RRT within seven days

**Fig. 5:**
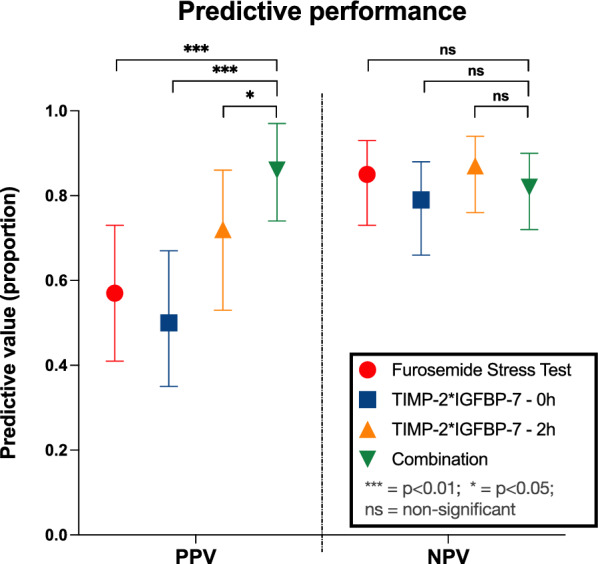
Positive Predictive Value (PPV) and Negative Predictive Value (NPV) of various assessment methods for predicting the need for RRT in SA-AKI patients. The combined assessment of upstream FST followed by TIMP-2*IGFBP-7 measurements two hours after the FST showed a significant improvement in PPV without a significant decrease in NPV, highlighting the utility of this approach in clinical decision-making

Integration of the FST and TIMP-2*IGFBP-7 measurements two hours post-FST in a logistic regression model demonstrated an AUROC of 0.84 (95%-CI: 0.75 to 0.93, supplementary file 1) for predicting RRT within seven days. Using the upstream FST followed by downstream TIMP-2*IGFBP-7 measurement (cutoff: 1.88 ng^2^/mL^2^/1000 according to Youden Index) after two hours to explore abnormal findings significantly improved the predictive accuracy (0.83; 95%-CI: 0.74 to 0.90) compared to the FST alone (accuracy 0.74; 95%-CI: 0.64 to 0.82; Fig. 3, 4). Consequently, we observed an increase of specificity to 0.96, and an increase to 0.86 in the positive predictive value (PPV) compared to the isolated FST (each p < 0.05, Table 3, Figs. 3, 4 and 5). The benefit of the combined assessment compared to the isolated FST is also reflected in the NRI. Our combined approach correctly reclassified 82.3% of the false-positive patients, resulting in a category-free NRI of 60.6% (95% CI 0.35 to 0.85, p < 0.01, supplementary file 2). Later measurements at four hours, six hours, and twelve hours did not differ in predictive performance compared to the results at two hours after FST (supplementary file 3).

### Univariate and adjusted multivariable logistic regression for the prediction of RRT within seven days

The combination of FST and TIMP-2*IGFBP-7 measurements after two hours, as well as the SOFA Score, urine output and lactate concentration were each associated with a greater risk of eventually receiving RRT within the next seven days in an univariable analysis (each p < 0.05). Here, the combined approach of FST and subsequent TIMP-2*IGFBP-7 revealed the highest odds ratio with a value of 27.43 (95%-CI: 8.00 to 129.21, p < 0.01). Upon considering all pertinent variables, a multivariable examination was performed. This unveiled that the combined approach of FST and TIMP-2*IGFBP-7 after two hours still remained an independent risk predictor for RRT within the subsequent seven days in SA-AKI, with an odds ratio of 12.19 (95%-CI: 2.44 to 81.16, p < 0.01), as illustrated in Table 4.

**Table 4:** Uni- and multivariate regression for dependency on RRT within the first 7 days

	Variable		RRT	Non-RRT	OR (univariable)	OR (multivariable)
Base characteristics	Age, years	Mean (SD)	67.7 (± 14.1)	68.6 (± 14.5)	1.00 (0.97–1.03, p = 0.758)	
	SOFA Score, Day 1	Mean (SD)	11.6 (± 2.6)	9.5 (± 2.8)	1.35 (1.13–1.63, p = 0.001)	1.31 (1.05–1.69, p = 0.025)
	Body Mass Index, kg/m^2^	Mean (SD)	27.5 (± 6.2)	27.3 (± 4.8)	1.01 (0.92–1.09, p = 0.892)	
	Urine output, mL, Day 1	Mean (SD)	1636.8 (± 2165.2)	4599.3 (± 2753.3)	1.00 (1.00–1.00, p < 0.001)	1.00 (1.00–1.00, p = 0.089)
Laboratory variables	Creatinine, mg/dL	Mean (SD)	2.3 (± 1.1)	2.5 (± 1.1)	0.90 (0.60–1.31, p = 0.584)	
	Potassium, mmol/L	Mean (SD)	4.6 (± 0.8)	4.5 (± 0.7)	1.26 (0.71–2.25, p = 0.434)	
	Urea, mg/dL	Mean (SD)	76.2 (± 50.0)	88.7 (± 44.2)	0.99 (0.98–1.00, p = 0.211)	
	CRP, mg/L	Mean (SD)	106.5 (± 98.7)	140.0 (± 106.6)	1.00 (0.99–1.00, p = 0.179)	
	PCT, ng/mL	Mean (SD)	19.9 (± 44.1)	12.6 (± 20.5)	1.01 (0.99–1.03, p = 0.339)	
	Lactate, mmol/L	Mean (SD)	3.3 (± 1.9)	2.1 (± 1.2)	1.72 (1.27–2.44, p = 0.001)	1.34 (0.90–2.03, p = 0.147)
TIMP-2*IGFBP-7 Measurements	TIMP-2*IGFBP-7 (ng^2^/mL^2^/1000) baseline	Low Risk, n (%)	12 (21.4)	44 (78.6)		
		High Risk, n (%)	19 (50.0)	19 (50.0)	3.67 (1.51–9.25, p = 0.005)	
	TIMP-2*IGFBP-7 (ng^2^/mL^2^/1000), 2 h after FST	Low Risk, n (%)	9 (13.4)	58 (86.6)		
		High Risk, n (%)	23 (71.9)	9 (28.1)	16.47 (6.07–49.51, p < 0.001)	
FST	FST (200 mL/2 h)	Low Risk, n (%)	9 (15.3)	50 (84.7)		
		High Risk, n (%)	23 (57.5)	17 (42.5)	7.52 (3.01–20.25, p < 0.001)	
Combined	FST (200 mL/2 h) and TIMP-2*IGFBP-7 (ng^2^/mL^2^/1000) after 2 h	Low Risk, n (%)	14 (17.9)	64 (82.1)		
		High Risk, n (%)	18 (85.7)	3 (14.3)	27.43 (8.00–129.21, p < 0.001)	12.19 (2.44–81.16, p = 0.004)

Classification into low and high-risk using TIMP-2*IGFBP-7 cut-off according to Youden-Index (as described in methods). Combined two-step approach consisting of an upstream Furosemide stress test (FST) with subsequent validation of abnormal finding via TIMP-2*IGFBP-7 measurements. Odds Ratio (OR) along with corresponding 95% confidence intervals

## Discussion

The study presents a promising approach for identifying high-risk SA-AKI patients requiring RRT within the first seven days of SA-AKI through combined testing using the FST followed by TIMP-2*IGFBP-7 measurements. Our combined approach demonstrates an intriguing specificity of 96% and enhances prediction for the need of RRT initiation. Thus, integration of a functional test along with a subsequent measurement of renal biomarkers in urine provided predictive enrichment. This may serve as a pragmatic strategy to identify high-risk SA-AKI patients who might benefit from early RTT initiation.

### Challenges in early identification of patients requiring RRT

Early identification of sepsis patients who may require RRT in their further course is a complex and yet unsolved challenge. Our cohort revealed only subtle initial differences in values of conventional variables between patients eventually requiring RRT and those managed with diuretics alone, highlighting the difficulties in discerning RRT candidates solely based on past medical history, renal function, concentrations of clinical chemistry variables, or disease severity (e.g., SOFA score). This emphasizes the need for investigations that provide clinicians with more objective information, i.e., such as those based on FST and urine biomarker concentrations.

### Diagnostic precision enhancement

In our cohort, as reported previously [14], the FST was a sensitive indicator of RRT. Nevertheless, the isolated reliance on FST alone would have led to a large number of patients unnecessarily undergoing RRT due to insufficient accuracy and low specificity (Table 3, Fig. 3) [20]. In total, 17 patients (out of 40 patients with a pathological FST) were able to achieve a negative fluid balance without RRT during the course of treatment.

Regarding TIMP-2*IGFBP-7, our pre-FST measurements showed a median of 1.90 (IQR 0.79 to 3.75) ng^2^/mL^2^/1000 and were thus in line with the recent literature, where concentrations ranged widely from 0.51 to 3.71 ng^2^/mL^2^/1000 in AKI and SA-AKI patients [31–33]. However, like the FST, measurements of TIMP-2*IGFBP-7 alone did not achieve sufficient accuracy to warrant the initiation of RRT. This was consistent regardless of whether conducted before, as demonstrated in previous studies [34, 35], or after the FST, where to date no data of TIMP-2*IGFBP-7 trajectories existed.

Finally, using the TIMP-2*IGFBP-7 measurements after FST and adopting a combined approach, i.e., an upstream preselection with FST followed by the determination of urinary TIMP-2*IGFBP-7 concentrations two hours after FST to also address tubular injury, we observed a notable improvement in diagnostic precision. This combined method not only significantly enhanced accuracy, reaching up to 83%, but also resulted in a robust specificity of 96% (Table 3). Additionally, there was an increase in the PPV to 86% without a significant loss in NPV (Fig. 5), as underscored by a significant NRI (supplementary file 2).

### Urinary biomarkers and the impact of furosemide

Following furosemide administration, a notable decrease in urine TIMP-2*IGFBP-7 concentrations compared to baseline was observed, suggesting potential dilution of biomarkers by increased urine volume. Interestingly, urine biomarker concentrations showed significant reductions both in the RRT and non-RRT subgroups that persisted over time. However, concentrations in RRT patients began to increase again from lower concentrations four hours post-furosemide, unlike the trajectory in non-RRT patients. This likely hints at underlying differences in renal injury between RRT and non-RRT patients unmasked by loop diuretics.

Regarding the prediction of RRT requirement, the effects of potential biomarker dilution due to a furosemide-induced increase in urine output were examined by multivariate analysis. We confirmed the combined TIMP-2*IGFBP-7 and FST's independent predictive power for RRT necessity despite adjustment for urine output. Taken together, these observations suggest that the behaviour of urine biomarker concentrations contains more information and is not solely explained by biomarker dilution due to increased urine output (Table 4).

In line with the literature [36], our work provides evidence for a relationship between the urinary biomarkers and diuresis. Increasing diuresis (as by a high-dose furosemide bolus) led to an improvement of predictive performance. Our finding of decreasing urinary TIMP-2*IGFBP-7 concentrations after furosemide also has implications for established cut-offs in the literature [37–39]. To achieve a high specificity in our study, we determined a cut-off of 1.88 ng^2^/mL^2^/1000 using the Youden Index. This number corresponds very closely with the manufacturer's cut-off for a high specificity (2.0 ng^2^/mL^2^/1000 for the early diagnosis of AKI [37]) and provided comparable predictive performance (supplementary file 4). However, depending on the defined cut-off for the downstream TIMP-2*IGFBP-7 test, our combined approach by aiming for high sensitivity can also favor the identification of patients for whom a "watch and wait" procedure might be advantageous. Notably, to increase sensitivity (e.g., sensitivity > 0.9) within the cohort with pathological FST results, a cut-off of 0.33 ng^2^/mL^2^/1000 would be required, which is close to the manufacturer's proposed cut-off for high sensitivity (i.e., 0.3 ng^2^/mL^2^/1000 for AKI) [27, 37, 38] (supplementary file 5). Nevertheless, cut-offs for urine concentrations of biomarkers under forced diuresis (high-dose diuretic administration), as elaborated in our study, will need further external validation.

### Timing of TIMP-2*IGFBP-7 measurements

We deliberately chose the 2-h time point for TIMP-2*IGFBP-7 measurements in our regression analyses to seamlessly integrate our approach into the ICU routine where efficient resource management is crucial [40]. Here, simultaneous evaluation of both the FST result and urine sampling for TIMP-2*IGFBP-7 measurements two hours after application of furosemide seems advisable. However, our measurements extending up to twelve hours following the diuretic bolus also revealed a consistently strong performance, indicating some flexibility in the timing of urine biomarker concentration measurements (supplementary file 3). Nonetheless, no additional prognostic benefit was derived from serial measurements, suggesting that a single TIMP-2*IGFBP-7 measurement after FST is sufficient from both clinical and economic perspectives.

### SA-AKI and long-term follow-up

On the prognostic side, our follow-up revealed an increased incidence of MAKE among SA-AKI patients necessitating RRT after 360 days, as shown in Table 2. This finding is particularly interesting considering the absence of significant differences in short term outcomes like ICU length of stay, 30-day mortality, or MAKE30 [41]. This observation hence highlights the necessity for a comprehensive, multidisciplinary care strategy extending beyond the typical 30-day follow-up period post-ICU discharge, particularly for SA-AKI patients with long-term risks. The results of our follow-up advocate for ongoing monitoring and collaboration among healthcare professionals to address the increased incidence of adverse kidney outcomes and dialysis dependency, and signal the critical need for further research and tailored aftercare programs to improve the prognosis of this vulnerable group [41, 42].

### Limitations

A limitation of our study is the relatively small sample size for a biomarker study. This may impact the generalizability of our findings and suggests that further research with larger cohorts is needed to confirm our results. It is important to note that the cut-off values we developed for the subsequent analyses of accuracy, PPV, and NPV are specific to our sample. Therefore, the generalizability of these cut-offs must be proven in future studies. While our study design and interpretation benefit from prospective recruitment, the inclusion criterion of “indication for negative fluid balance” may vary between individual patients and may have been influenced by the clinical judgment of the attending physicians involved. This ambiguity could lead to slight differences in the timing of enrollment or, despite somewhat aligned institutional standards, the timing of RRT initiation. However, upon assessment of later endpoints, such as MAKE 30, we were unable to discern a significant bias evoked by initial variations in the timing of enrollment. In addition, our findings may have improved by more frequent measurements of urine output and biomarkers, so as to assess a potential initial washout of biomarkers and their subsequent dilution by increased diuresis. Finally, our research does not yet extend to evaluate the potential therapeutic benefits and impact of our predictive enrichment scheme on patient outcomes. This aspect warrants further exploration in subsequent prospective randomized controlled trials. Ultimately, it should be noted that the use of TIMP-2*IGFBP-7 has been approved primarily for the early detection of AKI, and its use for predicting RRT so far remains an off-label application but is subject of investigations [21].

## Conclusion

Early and precise identification of SA-AKI patients who eventually require RRT remains challenging. The combination of an upstream FST with a single subsequent measurement of the urinary biomarkers TIMP-2*IGFBP-7 two hours after FST provided predictive enrichment regarding the necessity of dialysis. This approach significantly increased accuracy and achieved a specificity exceeding 95%. Next, it should be assessed, if patients identified by our predictive approach actually benefit from early initiation of RRT.

### Supplementary Information

Supplementary file 1.

Supplementary file 2.

Supplementary file 3.

Supplementary file 4.

Supplementary file 5.

## Data Availability

The data from this study are available upon reasonable request from the corresponding author.

## Abbreviations

AKI: Acute kidney injury
AUC: Area under the curve
AUROC: Area under the receiver operating characteristic curve
CI: Confidence interval
FST: Furosemide stress test
IGFBP-7: Insulin-like growth factor-binding protein 7
IQR: Interquartile range
KDIGO: Kidney disease improving global outcomes
LOS: Length of stay
MAKE: Major adverse kidney events
NRI: Net reclassification improvement
RCT: Randomized-controlled trials
ROC: Receiver operating characteristics
RRT: Renal replacement therapy
SA-AKI: Sepsis-associated acute kidney injury
SD: Standard deviation
TIMP-2: Tissue inhibitor of metalloproteinases 2
