# Supplementary material for: Predictive enrichment for the need of renal replacement in sepsis-associated acute kidney injury: combination of furosemide stress test and urinary biomarkers TIMP-2 and IGFBP-7
Source: Ann Intensive Care. 2024 Jul 13;14:111. doi: 10.1186/s13613-024-01349-4 (PMC11246358; doi:10.1186/s13613-024-01349-4)
Supplement: Supplementary file 1 [file 13613_2024_1349_MOESM1_ESM.pdf]

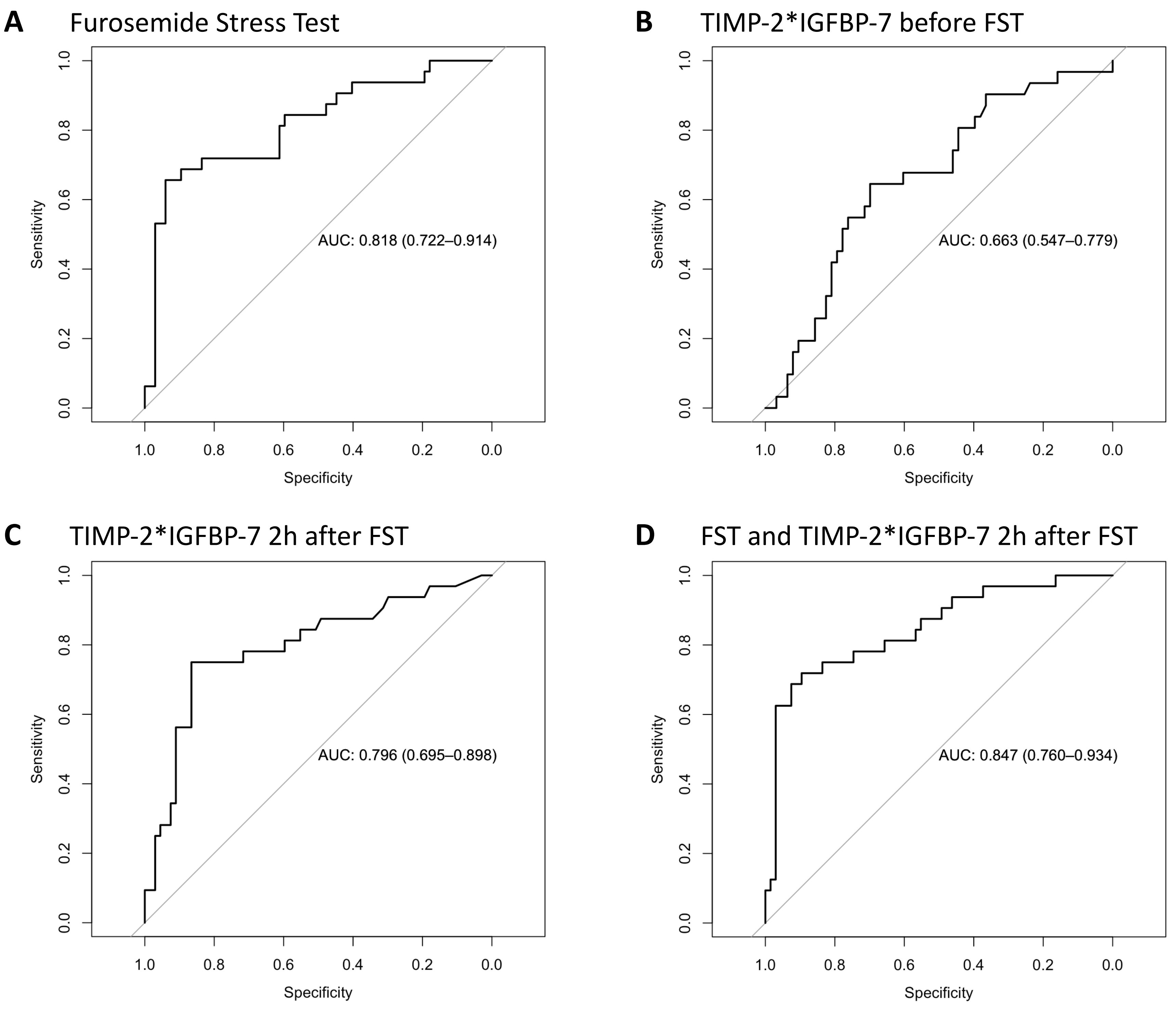
A
Furosemide Stress Test
AUC: 0.818 (0.722–0.914)
B
TIMP-2*IGFBP-7 before FST
AUC: 0.663 (0.547–0.779)
C
TIMP-2*IGFBP-7 2h after FST
AUC: 0.796 (0.695–0.898)
D
FST and TIMP-2*IGFBP-7 2h after FST
AUC: 0.847 (0.760–0.934)
Sensitivity
Specificity
0.0
0.2
0.4
0.6
0.8
1.0
