# Supplementary material for: Predictive enrichment for the need of renal replacement in sepsis-associated acute kidney injury: combination of furosemide stress test and urinary biomarkers TIMP-2 and IGFBP-7
Source: Ann Intensive Care. 2024 Jul 13;14:111. doi: 10.1186/s13613-024-01349-4 (PMC11246358; doi:10.1186/s13613-024-01349-4)
Supplement: Supplementary file 2 [file 13613_2024_1349_MOESM2_ESM.docx]

# Supplementary file 2: Net reclassification improvement of TIMP-2*IGFBP-7 Measurements 2 hours after FST

| **Net reclassification improvement** | **Value** | **95%-CI** | **p-value** |
| --- | --- | --- | --- |
|  |  |  |  |
| Category-free NRI (%) | 60.6 | 34.6 to 83.6 | <0.001 |
| % of non-RRT patients correctly reclassified | 82.3 | 64.0 to 100.0 |  |
| % of RRT incorrectly reclassified | 21.7 | -39.4 to -0.05 |  |
