# Supplementary material for: Predictive enrichment for the need of renal replacement in sepsis-associated acute kidney injury: combination of furosemide stress test and urinary biomarkers TIMP-2 and IGFBP-7
Source: Ann Intensive Care. 2024 Jul 13;14:111. doi: 10.1186/s13613-024-01349-4 (PMC11246358; doi:10.1186/s13613-024-01349-4)
Supplement: Supplementary file 3 [file 13613_2024_1349_MOESM3_ESM.docx]

Supplementary file 3: Test Accuracies of FST and TIMP-2*IGFBP-7 measurements at different time points

| **Test** | **Cut-offs** | **Accuracy** | **Sensitivity** | **Specificity** | **PPV** | **NPV** |
| --- | --- | --- | --- | --- | --- | --- |
| FST **and**  TIMP-2*IGFBP-7 **after 2h** | > 200mL/2h *and* > 1.88 ng^2^/mL^2^/1000 | 0.83  (0.74-0.90) | 0.56  (0.38-0.74) | 0.96  (0.88-0.99) | 0.86  (0.64-0.99) | 0.82  (0.72-0.90) |
| FST **and**  TIMP-2*IGFBP-7 **after 4h** |  | 0.82  (0.73-0.89) | 0.52  (0.33-0.71) | 0.96  (0.87-0.99) | 0.83  (0.59-0.96) | 0.82  (0.72-0.90) |
| FST **and**  TIMP-2*IGFBP-7 **after 6h** |  | 0.82  (0.73-0.89) | 0.53  (0.34-0.82) | 0.95  (0.87-0.99) | 0.84  (0.60-0.99) | 0.82  (0.71-0.90)) |
| FST **and**  TIMP-2*IGFBP-7 **after 12h** |  | 0.82  (0.73-0.89) | 0.53  (0.34-0.72) | 0.95  (0.87-0.99) | 0.84  (0.60-0.97) | 0.82  (0.71-0.90) |
