# Supplementary material for: Predictive enrichment for the need of renal replacement in sepsis-associated acute kidney injury: combination of furosemide stress test and urinary biomarkers TIMP-2 and IGFBP-7
Source: Ann Intensive Care. 2024 Jul 13;14:111. doi: 10.1186/s13613-024-01349-4 (PMC11246358; doi:10.1186/s13613-024-01349-4)
Supplement: Supplementary file 4 [file 13613_2024_1349_MOESM4_ESM.docx]

Supplementary file 4: Test Accuracy of Furosemide Stress Test (FST) and TIMP-2*IGFBP-7 measurements using Cut*-*off *according to manufacturer*

| **Test** | **Cut-off** | **Accuracy** | **Sensitivity** | **Specificity** | **PPV** | **NPV** |
| --- | --- | --- | --- | --- | --- | --- |
| FST **and**  TIMP-2*IGFBP-7 **after 2h** | > 200mL/2h *and* **> 2.00** ng^2^/mL^2^/1000 | 0.80  (0.71-0.87) | 0.52  (0.32-0.65) | 0.96  (0.87-0.99) | 0.83  (0.59-0.96) | 0.79  (0.69-0.96) |
| FST **and**  TIMP-2*IGFBP-7 **after 2h** | > 200mL/2h *and* **> 1.88** ng^2^/mL^2^/1000 | 0.83  (0.74-0.90) | 0.56  (0.38-0.74) | 0.96  (0.88-0.99) | 0.86  (0.64-0.97) | 0.82  (0.72-0.90) |

Combined evaluation of TIMP-2*IGFBP-7 and FST. Accuracy, sensitivity/specificity, positive predictive value (PPV) and negative predictive value (NPV), along with corresponding 95% confidence intervals, depending on cut-off value according to manufacturer (2.00 ng^2^/mL^2^/1000) or Youden-Index (1.88 ng^2^/mL^2^/1000)
