# Supplementary material for: Predictive enrichment for the need of renal replacement in sepsis-associated acute kidney injury: combination of furosemide stress test and urinary biomarkers TIMP-2 and IGFBP-7
Source: Ann Intensive Care. 2024 Jul 13;14:111. doi: 10.1186/s13613-024-01349-4 (PMC11246358; doi:10.1186/s13613-024-01349-4)
Supplement: Supplementary file 5 [file 13613_2024_1349_MOESM5_ESM.pdf]

Supplementary file 5:

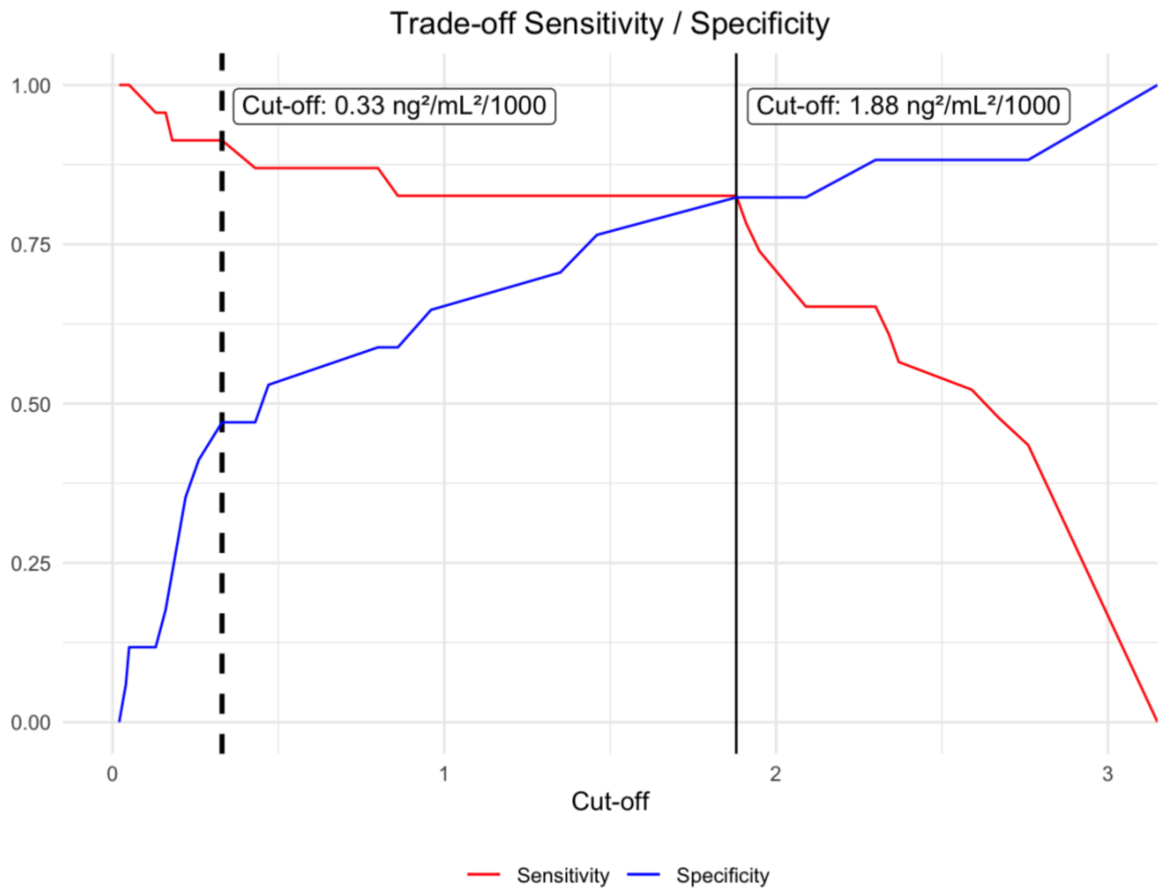

Figure Legend: Relationship between sensitivity and specificity for the cohort of patients with pathological FST results based on different cut-offs. The dashed line represents the cut-off needed to achieve a sensitivity of >90% while the solid line indicates the cut-off as determined by the Youden index.
